# Digitalization in Cranial Reconstruction: Revolutionizing Precision and Innovation

**DOI:** 10.7759/cureus.60046

**Published:** 2024-05-10

**Authors:** Shruti Deshmukh, Sweta G Pisulkar, Surekha A Dubey, Arushi Beri, Akansha Bansod

**Affiliations:** 1 Department of Prosthodontics, Sharad Pawar Dental College and Hospital, Datta Meghe Institute of Higher Education and Research, Wardha, IND; 2 Department of Prosthodontics and Crown and Bridge, Sharad Pawar Dental College and Hospital, Datta Meghe Institute of Higher Education and Research, Wardha, IND; 3 Department of Prosthodontics, Sharad Pawar Dental College and Hospital, Acharya Vinoba Bhave Rural Hospital, Wardha, IND

**Keywords:** digitalization, virtual surgical planning (vas), 3d bioprinting, 3d reconstruction, cranial defects

## Abstract

Cranioplasty for cranial defects can be complex and challenging in composite defects. The intricate 3D structure of the craniofacial skeleton poses various difficulties encountered in surgical reconstruction. The continuous progress in computer-aided design and computer-aided manufacturing design, and fabrication technology has led to a growing array of applications for visual analog scale and 3D printing in craniofacial surgery, encompassing preoperative assessment, the creation of cutting guides, and the development of custom implants and stereolithographic models. Within this review, the authors detail the present and developing applications of virtual surgical planning, 3D bioprinting, augmented reality, and virtual reality in craniofacial reconstruction.

## Introduction and background

Cranial abnormalities can arise from a variety of causes, including degenerative disorders, surgery, trauma, and congenital and acquired conditions. Between one in 2,000 and one in 2,500 live births is the prevalence of craniosynostosis, and this incidence has risen over time. Predisposing variables include genetic alterations as well as environmental factors such as maternal smoking, fetal posture, intrauterine constraint, and exposure to teratogens during pregnancy. About 20% of cases of craniosynostosis are due to genetic factors, which are frequently inherited in an autosomal dominant way and result in new mutations in 50% of cases. Syndromic craniosynostosis accounts for 25% of cases, while nonsyndromic craniosynostosis accounts for 75% of cases [1].

Cranial abnormalities can potentially result from factors like congenital anomalies, neurosurgery procedures such as decompressive craniotomies, tumor excisions, trauma, and infections. For these problems, cranioplasty is used as a treatment to protect the underlying brain tissues. Furthermore, cranioplasty improves the appearance and relieves pain, which eventually raises the quality of life. Small flaws that are 2-3 cm in size and located just above the orbital rim or nasion may require repair for aesthetic purposes [2]. Conversely, defects exceeding 8 cm in size at the posterior parieto-occipital junction may require comprehensive repair primarily for brain protection. Cranioplasty is indicated for various reasons, including addressing social stigma resulting from disfigurement, providing mechanical protection, alleviating pain associated with postsurgical cranial defects, and managing defects that are not amenable to alternative surgical interventions [3].

Osteoplastic reconstruction and alloplastic implantation are the two techniques used in cranial reconstruction. Neurosurgeons frequently choose decompressive craniectomy (DC) combined with duraplasty in cases of traumatic brain injury with potentially fatal elevated intracranial pressure. The advantages of DC are logically supported by the worldwide documentation [4]. Alloplastic materials, autogenous bone, such as metals, polyethylene, and silicone, and heat-polymerizing acrylic are often utilized materials for cranial reconstruction. Polyether ether ketone was recently introduced for cranioplasty and has proven to be an effective implant material in orthopedics. A more modern material called titanium has been used to make cranial prosthetics [5].

The use of computer-assisted surgery (CAS) offers several benefits compared to the conventional free-hand technique, resulting in more efficient and reliable outcomes in reconstruction. Virtual surgical planning (VSP) creates a digital platform that enables surgeons to anticipate, predict, and proactively address surgical complications. The implementation of VSP utilizing computer-aided design and computer-aided manufacturing (CAD-CAM) techniques has created a different workflow to enhance the precision of preoperative planning, minimizing the reliance on trial and error during surgery. The integration of digital technologies into modern practices has greatly improved the standard of maxillofacial rehabilitation [5].

CAS is a four-stage reconstructive surgery procedure. The initial phase involves a visual analog scale (VAS), followed by 3D modeling. The third phase includes the intraoperative transfer of the virtual design or the surgical phase. The concluding stage of CAS encompasses postoperative evaluation, which is often overlooked despite its integral role [6].

## Review

Digital imaging in cranial reconstruction

The introduction of VSP using CAD-CAM techniques has provided an effective option for more accurate preoperative planning, reducing the need for trial and error during surgery. This involves preoperative planning by creating cutting guides and bone models through virtual surgery fabrication using stereolithography (SLA) techniques, as well as using surgical navigation systems to assist in implant placement and guide bone cuts [7].

**Figure 1:**
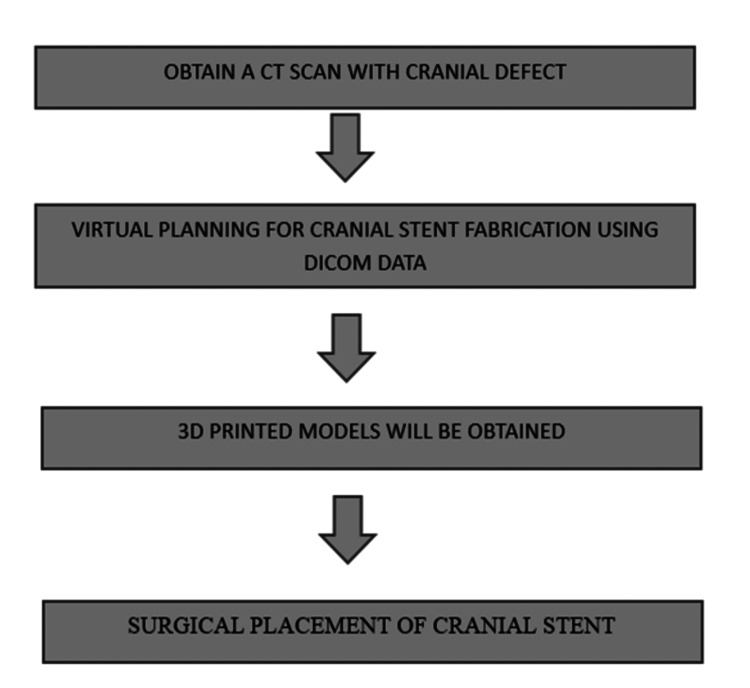
Workflow for digital scanning of cranial defect Figure credits: Shruti Deshmukh

VSP has been proven effective in assisting with craniosynostosis reconstruction, offering reproducible and objective results that enhance overall outcomes. This approach provides surgeons with a clear understanding of the outcome before the surgery commences. Additionally, the VSP process enables relatives to comprehend the pathology and surgical steps through visual 3D images. During surgical planning, the surgeon can precisely visualize the location of planned cuts relative to crucial structures around the brain, thus mitigating potential complications. Throughout the planning phase, the bioengineer presents axial and coronal cuts corresponding to the 3D model, illustrating where these cuts will impact the structures around the skull base and brain. To ensure accurate planning, the utilization of VSP mandates a CT scan within one or two months of the scheduled surgery. In this method, first, we will obtain a CT scan of the cranial defect, which will accurately depict the size and extent of the defect. CT scan is a major requirement for VSP. Once this is done, then we need to do VSP for cranial stent fabrication using Digital Imaging and Communications in Medicine (DICOM) data. Then, these CT images and the DICOM file are merged to get a 3D-printed model. After that, surgery is performed to place the cranial stent. Figure 1 shows the workflow for digital scanning of cranial defects [8].

Methods of 3D Printing

3D printing techniques are used in additive manufacturing, which utilizes CAD software to construct 3D objects by sequentially layering various materials. Moreover, 3D printing encompasses multiple production technologies, including solid methods (such as fused deposition modeling [FDM]), powder-based approaches (such as selective laser sintering [SLS] and selective laser melting), as well as liquid-based methods (such as SLA and digital light processing). Among these, SLA stands out due to its superior speed, controlled integrity, high-print resolution, and the absence of heat during the printing process [9].

3D printing has revolutionized cranial reconstruction in several ways, offering numerous advantages. One of the biggest advantages of 3D printing in cranial reconstruction is the ability to create customized implants tailored to each patient's unique anatomy. This customization ensures a better fit and improves overall outcomes. 3D printing allows for the creation of highly precise and accurate cranial implants based on digital scans of the patient's skull. This reduces the margin of error during surgery and enhances the overall safety of the procedure.

Furthermore, customized 3D-printed implants often require less intraoperative modification, leading to reduced surgery time. This can contribute to faster patient recovery times and lower healthcare costs. Many 3D printing materials used in cranial reconstruction, such as titanium alloys and medical-grade polymers, are biocompatible. They integrate well with the patient's tissues, reducing the risk of complications such as rejection or infection.

Also, one of the major advantages of 3D printing is its patient-specific design. Practitioners can collaborate with biomedical engineers to design implants that precisely match the missing or damaged cranial structures of individual patients. This promotes better aesthetic outcomes and functional restoration. 3D printing also enables the fabrication of complex cranial structures that would be challenging or impossible to create using traditional manufacturing methods. This is particularly beneficial for cases involving intricate cranial defects or reconstructions. While initial setup costs for 3D printing can be significant, the technology can ultimately be cost-effective for cranial reconstructions. It reduces the need for extensive manual labor and multiple surgeries due to better fit and functionality.

Virtual Reality and Augmented Reality

Virtual reality (VR) entails immersing the user within a system that blocks out the natural world, creating a virtual environment for the user to experience. Augmented reality (AR), in contrast to VR, involves a blending of elements from the natural and virtual worlds. In AR, virtual images are superimposed onto the real-world environment. This allows the user to directly view virtual planning on the patient, facilitating simultaneous perception and interaction between the projected virtual image and the natural environment. VR has gradually gained importance in neurosurgery [10].

These platforms enable multidisciplinary teams, including surgeons, radiologists, and biomedical engineers, to collaborate more effectively. They can review and discuss surgical plans in a shared virtual environment, fostering better communication and decision-making. VR and AR can be used to create interactive educational experiences for patients undergoing cranial reconstruction. Patients can visualize their condition, understand surgical procedures, and make informed decisions about their treatment options. This will ultimately help the operator in carrying out proper treatment. Hence, this can be a very beneficial tool in the field of cranial reconstruction.

3D Bioprinting

Utilizing bioinks, 3D bioprinting employs an additive manufacturing process to construct structures layer-by-layer, mimicking the behavior and structures of natural tissues by incorporating living cells. The bioinks, constituting the material in bioprinting, consist of natural or synthetic biomaterials that can be combined with living cells. Consequently, the technology of 3D bioprinting can be applied to produce a cranioplasty material laden with cells to facilitate bone regeneration. An autologous bone scaffold is created for cranioplasty using 3D bioprinting technology. To replicate the structures of the skull, an external lamina is designed using polycaprolactone, and the 3D-printed autologous bone, along with a hydrogel containing bone marrow-derived mesenchymal stem cells (BMSCs), is employed to mimic cancellous bone for bone regeneration. The scaffold demonstrates exceptional cellular affinity, fostering osteogenic differentiation of BMSCs in both 2D and 3D culture systems. Subsequently, the scaffold is implanted in cranial defects in beagle dogs for up to nine months, leading to the promotion of new bone and osteoid formation. Transplanted BMSCs further differentiate into vascular endothelium, cartilage, and bone tissues, while native BMSCs are recruited into the defect. This offers a method for bedside bioprinting of a cranioplasty scaffold for bone regeneration, presenting an avenue for future clinical applications of 3D printing [11].

One of the greatest advantages of 3D bioprinting technology is that it can fabricate complex tissue structures, including bone-like materials, cartilage, and even neural tissues. This bioprinted model can stimulate tissue regeneration and healing processes due to the presence of living cells and bioactive factors within the constructs. This regenerative potential is beneficial for promoting bone growth, vascularization, and tissue remodeling in cranial reconstruction. Also, bioprinted cranial stents can be designed to resist infections better than traditional implants. Incorporating antimicrobial agents or designing structures that discourage bacterial adhesion can help reduce the risk of postoperative infections. Ongoing research in bioprinting continues to explore novel biomaterials, printing techniques, and bioink formulations, promising further advancements in cranial reconstruction outcomes, including improved implant durability and functionality.

Discussion

The neurosurgical procedure of cranioplasty involves the use of cranial prostheses crafted from materials like titanium, autologous bone, ceramics, and polymers. Cranioplasty can be done with conventional methods that include the fabrication of polymethylmethacrylate (PMMA) cranial stents by mold formation. However, this method can be difficult if a patient has neuromuscular incoordination and also when margins of the defect cannot be accurately detected; hence, a better and more accurate method for cranial reconstruction is needed [12-15]. Through emerging technology and newer concepts in this field, perfect cranial symmetry is attained with minimal intraoperative adjustments, ensuring precise coaptation with the help of 3D printing and VAS [16]. Various 3D-printing technologies like FDM, SLA, and selective laser sintering (SLS) are extensively used for craniofacial reconstruction. Additionally, unlike PMMA prostheses generated intraoperatively, there is no exothermal reaction due to gas formation, thereby avoiding polymerization heat and minimizing adjustment time. The utilization of VSP has emerged as a game changer in cranial reconstruction. Surgeons can now simulate procedures in a virtual environment, allowing for meticulous preoperative planning. VSP enables the customization of surgical approaches based on individual patient anatomy, optimizing outcomes and minimizing risks [17].

The digitalization of cranial reconstruction represents a paradigm shift in neurosurgical practices. From advanced diagnostic imaging and VSP to 3D printing and telemedicine, the integration of digital technologies has enhanced precision, personalized care, and overall outcomes. As per the studies conducted in the field of 3D printing, it has been evaluated that the percentage error for PolyJet, SLS, and 3DP printers was 0.18%, 0.79%, and 0.67%, respectively; hence, we get maximum accuracy with 3D printing [18]. However, it is imperative to navigate the challenges of data security and ethical considerations to ensure the responsible and effective implementation of digital tools in cranial surgery. As technology continues to evolve, the future holds even more promise for further innovations in this dynamic and rapidly advancing field [19,20].

As digitalization becomes more ingrained in cranial reconstruction, concerns regarding data security, patient privacy, and ethical considerations come to the forefront. Efforts can be made to safeguard patient information further, ensure compliance with regulatory standards, and maintain ethical practices in the digital era of cranial surgery [21].

## Conclusions

The exploration of digitalization in cranial reconstruction, along with its applications and practicality, persists with the emergence of new technology and growing experience with current technology. The implementation of VSP can enhance the precision of craniofacial skeleton reconstruction. 3D bioprinting can significantly contribute to the development of cranial stents with increased accuracy. 3D-printing technologies offer benefits such as efficient material utilization and the ability to produce a singular intricate geometry. The future trajectory of 3D printing in prosthodontics will be shaped by the development of new materials and technologies, undeniably indicating a bright future for 3D printing. Also wit use of AR and VR patient education will become better and easy in such complex cases of cranial reconstruction. Essentially, the progress in the digitalization of cranial reconstruction is notable, promising a future where innovative technologies will continuously transform and improve surgical procedures, ultimately benefiting both healthcare professionals and the patients they serve.
